# Supplementary material for: Systemic immune-inflammation index as an independent risk factor for diabetic nephropathy: a retrospective, single-center study
Source: PeerJ. 2024 Nov 18;12:e18493. doi: 10.7717/peerj.18493 (PMC11639188; doi:10.7717/peerj.18493)
Supplement: Table S3 [file peerj-12-18493-s001.docx]

| **^coefficient^** | | | | | | | | |
| --- | --- | --- | --- | --- | --- | --- | --- | --- |
| Models | | Unstandardized Coefficients | | Standardized Coefficients | t | Sig. | Collinearity statistics | |
|  |  | B | Std. Error | Beta |  |  | Tolerance | VIF |
| 1 | (constant) | .863 | .146 |  | 5.901 | .000 |  |  |
|  | BUN | .010 | .008 | .119 | 1.335 | .184 | .314 | 3.180 |
|  | CR | .000 | .000 | -.026 | -.284 | .776 | .308 | 3.250 |
|  | UA | .001 | .000 | .143 | 2.506 | .013 | .778 | 1.286 |
|  | GFR | -.008 | .001 | -.580 | -8.327 | .000 | .520 | 1.925 |
| a. Dependent variable：DKD | | | | | | | | |

Collinearity statistics among codependent variables (uric acid, creatinine, eGFR and blood urea nitrogen). According to the results, with VIF < 5, it can be assumed that there is no collinearity between these variables.
