# Supplementary material for: Systemic immune-inflammation index as an independent risk factor for diabetic nephropathy: a retrospective, single-center study
Source: PeerJ. 2024 Nov 18;12:e18493. doi: 10.7717/peerj.18493 (PMC11639188; doi:10.7717/peerj.18493)
Supplement: Table S4 [file peerj-12-18493-s002.docx]

| **Hosmer-Lemeshow test** | | | |
| --- | --- | --- | --- |
| steps | chi-square | Degrees of freedom | significance |
| 1 | 8.167 | 8 | .417 |
| 2 | 32.397 | 8 | .000 |
| 3 | 25.872 | 8 | .001 |
| 4 | 1.738 | 8 | .988 |
| 5 | 5.899 | 8 | .659 |
| 6 | 4.027 | 8 | .855 |

The Hosmer-Lemeshow test was used to evaluate goodness of fit. The Hosmer-Lemeshow test suggests that the final model p-value is 0.855, which is greater than 0.05 and is considered to be a good fit.
